# Challenges in the Dental Management of Rett Syndrome under General Anesthesia: A Rare Disease

**DOI:** 10.1155/2022/4038221

**Published:** 2022-02-01

**Authors:** Wisam Al-Hathlol, Raed Bokhari, Nada Alzahrani, Elaf Alkuwaiti

**Affiliations:** ^1^Department of Pediatrics, National Guard Hospital, Dammam, Saudi Arabia; ^2^College of Dentistry, Imam Abdulrahman Bin Faisal University, Dammam, Saudi Arabia

## Abstract

Rett syndrome is a neurodevelopmental genetic X-linked disorder. It is predominantly found in females with a prevalence rate of 1 : 9000. Rett syndrome patients are usually healthy the first months of their lives. The syndrome goes into a deceleration phase where motor, behavioral, and cognitive skills are impaired. Regarding their oral health, bruxism is one of the common oral manifestations found among Rett syndrome patients. We present a case of an 8-year-old patient with Rett syndrome who presented to the dental clinic for oral rehabilitation. The patient was evaluated and treated under general anesthesia with multiple extractions, restorations, and crown installments. Oral rehabilitation of Rett syndrome is important for those patients, and proper evaluation and treatment are the most efficient when performed under general anesthesia.

## 1. Introduction

Rett syndrome (RS) is a rare neurological X-linked disorder caused by a dominant mutation in the MECP*2* gene, which exclusively affects females with a prevalence of 1 to 9000 (1, 2). Rett was the first to define the disease in 1966 (3), and Hagberg et al. provided comprehensive clinical characteristics of the syndrome in 1983 (4). In RS, patients are usually healthy for the first 6-18 months of life. Afterward, they begin to exhibit deceleration of head growth and muscular hypotonia which is followed by loss of motor coordination, acquired communication, and social skills (5). Furthermore, patients go through a period of stagnation between 2 and 10 years of age followed by a rapid regression in motor, behavioral, and cognitive skills. They are characterized by autistic-like behaviors such as mental retardation, hand tremors, and epileptic seizures. Other features including hyperventilation, apnea, kyphosis, development of osteoporosis, gastrointestinal problems, and weight loss can be found in RS (5–8). Moreover, around 50% of the affected patients may develop orthopedic deformities in late stages including scoliosis and/or multiple joint contractures as well as vascular and arterial malformations which can impede venous access (9).

RS patients can develop some oral manifestations including high arched palate, anterior open bite, bruxism, gingivitis, periodontitis, gingival hyperplasia, xerostomia, glossitis, erythema multiform, lingual paralysis, dental anomalies, and delayed eruption (10–14). Bruxism in RS is the most frequently observed oral habit, especially the diurnal bruxism that ranges from (53-95%) (15–17), followed by tongue thrusting (11), digits sucking (13), and oral breathing (11, 15). Many of the oral manifestations are linked with the anxiolytics and anticonvulsants therapies taken (10, 18).

RS diagnosis is mostly clinically based with no cure or specific treatment available to date (19, 20). Their management requires an interdisciplinary approach that covers pharmacological, symptomatic therapy, physiotherapy, social work, and speech therapy (21). Dental treatment and sedation used for such a complex syndrome differ. Some use nitrous oxide alone (22) or combined with sublingual midazolam (15), while some investigators prefer to use general anesthesia since the patients are considered at a high medical risk and are difficult to cooperate (10, 23). In this case report, we aim to stress the challenges in the dental management of RS during general anesthesia.

## 2. Case Report

An eight-year-old female patient with Rett syndrome was scheduled to undergo oral rehabilitation under general anesthesia. She came to the dental clinic complaining of several cavities and discomfort in the posterior teeth. Preoperative examination showed mental retardation, normal consciousness, and lack of cooperation and orientation. Stiffness of the lower extremities, inability to stand, and the need for support to sit were noted. The patient was diagnosed with Rett syndrome after having multiple seizures and stereotypic hand movement at the age of two and a genetic blood test confirmed the presence of MECP2 mutation. Regarding her medication, the patient was not on any medication and was using herbal medication only. Meanwhile, her clinical examination including cardiac, respiratory, and gastrointestinal was normal. Her hemoglobin level was 138 gm/L, and serum electrolytes were within normal limits.

**Figure 1:**
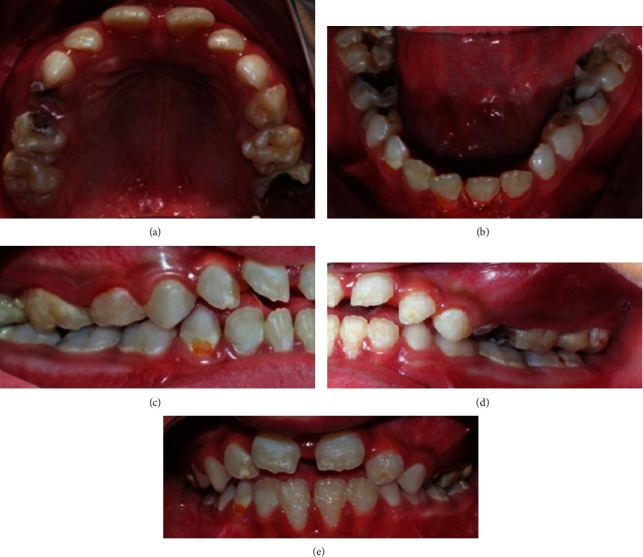
Intraoral preoperative photographs showing (a) maxillary occlusal view. (b) Mandibular occlusal view. (c) Right lateral view. (d) Left lateral view. (e) Frontal view.

In regard to her dental examination, extraoral examination was insignificant. The patient had symmetrical face, and the lips were incompetent and dry. On the other hand, her intraoral examination was remarkable. Soft tissue examination showed normal alveolar mucosa, macroglossia, with generalized gingivitis with no abnormality in the floor of the mouth. The hard tissue examination showed mixed dentition stage matching chronological age, generalized enamel hypomineralization, multiple carious teeth, malocclusion, and anterior open bite (Figure 1).

**Figure 2:**
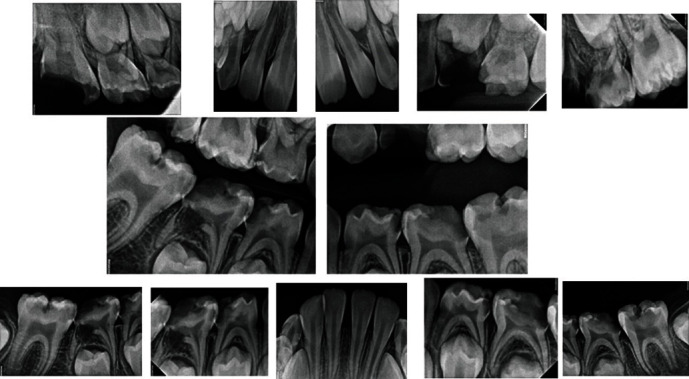
Full mouth preoperative radiographs.

Preoperatively, the patient received 1 mg of midazolam intravenously (IV). Regarding her vitals, her heart rate was 99/min, blood pressure was 112/64 mmHg, respiratory rate was 25/min, and peripheral oxygen saturation (SpO_2_) was 99%. Before starting the operation, an administration of 20 mcg/kg of fentanyl, 40 mg/kg propofol, remifentanil 10 mcg/kg, and 2 mg/kg of dexamethasone was given IV. During the operation, administration of 10 mcg/kg of fentanyl, 80 mcg/kg remifentanil, and 12.5 mg/kg of diclofenac sodium was added. After initiating of general anesthesia through nasotracheal intubation, an intraoral radiographs and photographs showed multiple caries in the primary and permanent teeth with some pulpal involvement (Figure 2).

**Figure 3:**
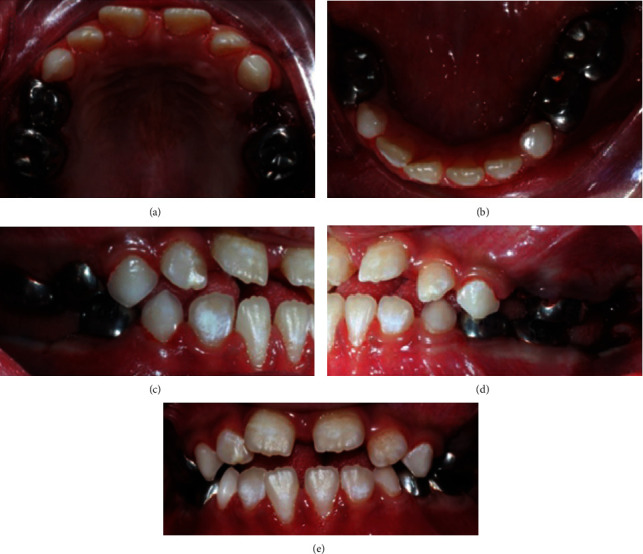
Intraoral postoperative photographs. (a) Maxillary occlusal view. (b) Mandibular occlusal view. (c) Right lateral view. (d) Left lateral view. (e) Frontal view.

**Figure 4:**
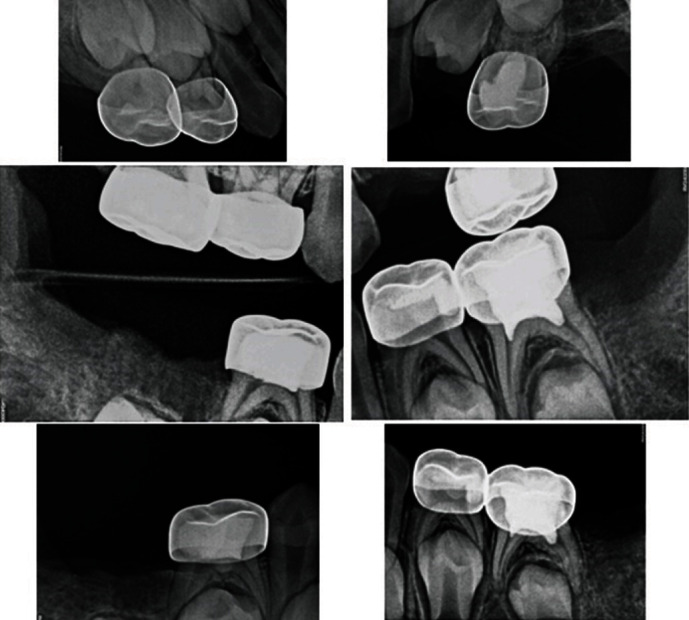
Postoperative radiographs.

Therefore, the treatment plan was set to extract the decayed permanent teeth #16, #26, #36, and #46 as well as #64 and #85 primary teeth. A formacrsol pulpotomy was performed on teeth #65, #75, and #84 followed by Resin modified glass ionomer filling. Afterwards, the occlusal seal was achieved using stainless steel crowns. Stainless steel crowns were a superior treatment choice on teeth #55, #54, and #74 due to the patient's high risk for caries. A buccal composite restoration was done on tooth #83 along with prophylaxis and topical fluoride (Figures 3 and 4).

The operation took three hours and 24 minutes. The patient was taken to the recovery room afterwards and was monitored until she regained full motor function. She was discharged the next day and was given acetaminophen 24 mg/ml, elixir (LASA) of 160 mg oral QID every 6 hours PRN for mild pain for a total of five days, penicillin (250 mg/5 ml) suspension BID, and q12h for a duration of five days. Her discharge instructions were given to brush the teeth twice daily, reduce sugar intake, and undertake a healthy diet with regular follow-ups in the clinic.

## 3. Discussion

Rett was the first to define the disease in 1966 (3). RS is a rare developmental X-linked disorder caused by a dominant mutation in the MECP*2* gene (16). However, this syndrome exclusively affects females with a prevalence of 1 to 9000 (2). The Rett syndrome is categorized in detective phases into an initial phase of early normal development, followed by a regression phase, appearing as severe intellectual disability. Moreover, another study categorized RS into four conventional phases starting from a delayed developmental milestone to further regression with loss of acquired communication and signs of mental retardation. These were followed by neuromotor challenges such as walking, followed by a later phase of motor deterioration which is characterized by weight loss and skeletal deformities (24). In our case, inability of cooperation and orientation was noted along with stiffness of the lower extremities, inability to stand, and the need for support to sit that were noted. Other researchers reported the most common oral findings were bruxism, masseteric hypertrophy, anterior open bite mouth breathing, tongue thrusting, digital sucking, and high arch palate (25). In our case, there were no clinical signs of attrition or masseteric hypertrophy, but there was nonnutritive sucking habit.

In 2018, Lai et al. reviewed the Australian Rett Syndrome Database (ARSD) which was established in 1993. The registry includes all female RS patients born since 1976. They found that around two third of the population of RS attended the dental clinic with slightly higher proportion with those having epileptic seizures. In addition, 75% of those with gastroesophageal reflux disease attended the dental clinic compared to 56% to those without the disease. Moreover, dental procedures that was done under general anesthesia were reported in 24 patients out of 77. It is worth mentioning that general anesthesia was performed on small procedures such as one tooth extraction. The authors also found that patient who underwent general anesthesia was reported higher in those living in inner regions compared to those living in major cities (26). This can be attributed to the epileptic, noncompliant nature of RS. In addition, proper evaluation would be performed more efficiently to diagnose any other dental disease. In our case, the patient underwent full investigatory panel under general anesthesia where multiple carries were discovered with pulp involvement.

According to Mahdi et al., the most common dental finding among RS patients is bruxism. Other findings include masseteric hypertrophy, anterior open bite, and nonphysiological tooth wear (16). This was also found in an older study by Magalhães et al. where they studied a total of 13 patients with RS and found that bruxism was the most common dental manifestation (27). However, our patient did not report bruxism or any masseteric hypertrophy. Meanwhile, Bianco and Rota stated that some extraoral manifestation may appear which are related to pharmacological therapy. These include xerostomia, glossitis, erythema multiforme, gingival hyperplasia, dysphagia, and lingual paralysis. In our patient, there were no oral manifestation present, however, she was not on any pharmaceutical therapy and was only treated by herbal medications.

## 4. Conclusion

Rett syndrome is a neurodevelopmental disorder which presents with a constellation of clinical findings. Based on the clinical representation, it is possible to diagnose a case of Rett syndrome. Early detection of the disease is important. A suitable multidisciplinary approach for management should be well established to improve the quality of life. Proper dental evaluation and management under general anesthesia are recommended due to the noncompliance nature of the patient.

## Data Availability

All the data is available within the text.
